# Catechin Mediates Ferroptosis to Exert an Anti-Inflammatory Effect on RAW 264.7 Cells

**DOI:** 10.3390/foods11111572

**Published:** 2022-05-27

**Authors:** Weiyang Kuang, Jiajia Yang, Zhiyuan Liu, Jinzi Zeng, Xuewei Xia, Xiaodan Chen, Saiyi Zhong, Riming Huang

**Affiliations:** 1 Guangdong Provincial Key Laboratory of Food Quality and Safety, College of Food Science, South China Agricultural University, Guangzhou 510642, China; 20212145014@stu.scau.edu.cn (W.K.); yangjiajia@stu.scau.edu.cn (J.Y.); aiden@stu.scau.edu.cn (Z.L.); zengjinzi1999@stu.scau.edu.cn (J.Z.); xiaxuewei@stu.scau.edu.cn (X.X.); 18125907913@163.com (X.C.); 2 College of Food Science and Technology, Guangdong Ocean University, Zhanjiang 524088, China; zhongsy@gdou.edu.cn

**Keywords:** catechin, anti-inflammatory, ferroptosis, molecular mechanism

## Abstract

Catechin possesses a potential anti-inflammatory activity, but its anti-inflammatory mechanism is still unclear. Herein, the analysis of network pharmacology showed that catechin might mediate ferroptosis on macrophages to exhibit a significant anti-inflammatory effect on RAW264.7. The metabolomics further indicated that catechin might influence ferroptosis by activating two pathways of cysteine and methionine metabolism and glutathione metabolism, and inhibiting the pathway of ferroptosis to promote the reduction of l-methionine-s-oxide and s-glutathionyl-l-cysteine, and the reduction and synthesis of γ-glutamylcysteine. Furthermore, related proteins (MSRA, CDR, GSR and GCL) in three metabolic pathways and ferroptosis-related proteins (GPX4 and SLC7A11) might be relevant to catechin through molecular docking. Thus, we speculate that catechin plays an anti-inflammatory effect through mediating ferroptosis on RAW264.7, which still needs further focus on the detailed molecular mechanism.

## 1. Introduction

Inflammation is an adaptive response of the body to harmful stimuli produced by the outside world or internal organism, such as infection and tissue injury [1]. It plays an essential role in taking part in the important physiological process of many diseases including cancer [2], diabetes [3], anemia [4], Alzheimer’s disease [5] and atherosclerosis [6]. This determines its complexity. Thus, the biological mechanism of inflammation has always been a challenge for researchers. In recent years, they have begun to try to analyze the occurrence of inflammation at the cellular and molecular levels [7]. Ferroptosis, a new type of cell death, has caught the attention of researchers with deeper investigation. They have found that ferroptosis is different from other types of cell death in morphology, biology and genetics and it has an inner connection with inflammation [8]. Ferroptosis belongs to non-apoptotic cell death which is also known as “regulatory necrosis”. It is an oxidative cell death driven by lipid peroxidation [8,9]. Lipid peroxidation can be caused by the inhibition or the destruction of cysteine-glutamate antiporter system XC-. This system is regulated by the solute carrier family 7 member 11 (SLC7A11) and the solute carrier family 3 member 2 (SLC3A2) [10]. Its limitation can cause the failure of antioxidant glutathione to be supplemented in time. This will increase the level of intracellular reactive oxygen species (ROS) [11]. As a result, ferroptosis is speculated to be a new target of inflammatory diseases. Some researchers have found that both ferroptosis and inflammatory diseases are related to the loss of glutathione peroxidase 4 (GPX4) and glutathione (GSH), the increase of lipid peroxidation products, and the disorder of iron metabolism [12]. However, the effects of ferroptosis on inflammation are still not clear. Whether ferroptosis is involved in inflammatory diseases, and whether inducing ferroptosis can achieve the purpose of promoting anti-inflammation, are questions that still need further exploration.

Catechin belongs to the flavonoid family which is also known as flavan-3-ols (or flavanols). It is a natural polyphenol compound that mainly exists in green tea, red wine, strawberries, black grapes and apricots [13]. The group of catechin in green tea include: (-)-epigallocatechin-3-gallate (EGCG), (-)-epicatechin-3-gallate (ECG), (-)-epigallocatechin (EGC) and (-)-epicatechin (EC) [14]. Catechin has a strong antioxidant capacity because there are many hydrocarbon groups in polyphenol molecules. Therefore, some researchers have begun to explore the active effects of catechin on related diseases due to its characteristics of being healthy, green, rich in sources, and strong in antioxidant capacity. Anti-inflammatory, antioxidant and chemopreventive activities have been considered as the most important effects of catechin [14]. In addition, catechin also has the functions of antiviral [15] and anticancer [16] and also plays a beneficial role in preventing major diseases such as diabetes, cardiovascular diseases and neurodegenerative diseases [13]. Thus, some researchers have conducted in-depth research on its specific molecular mechanism in recent years due to the excellent anti-inflammatory and antioxidant activities of catechin. They have found that an epicuticular wax (ECW) which is extracted from *Eugenia brasiliensis* Lam. (Myrtaceae) leaves has an anti-inflammatory activity due to the inhibition of the migration of inflammatory mediators. As a result, they speculated that this effect might be related to catechin and gallic catechin contained in ECW [17]. In addition, they have also explored the synergistic effects of catechin and other flavanols. They have found that the synergistic inhibitory effects of procyanidin B_2_ and catechin are better than the inhibitory effects of their single use on acrylamide. Acrylamide is a carcinogenic and toxic substance and will cause oxidative damage and systemic inflammation [18,19]. It is worth noting that catechin is associated with ferroptosis and this relevance has still not been found in other flavanols. A study has shown that green tea derivative EGCG can activate the nuclear factor erythroid 2-related factor 2 (Nrf2) signaling pathway to eliminate ROS, inhibit apoptosis and ferroptosis, and improve the intestinal injury caused by ionizing radiation [20]. However, whether ferroptosis is involved in the anti-inflammatory process of catechin has not been reported, and its specific mechanism has still not been clarified.

In this study, we preliminarily verified the cytotoxicity and anti-inflammatory activity of catechin. Secondly, the analysis of network pharmacology and metabolomics were integrated to clarify the anti-inflammatory capability of catechin mediated ferroptosis on RAW264.7 according to the results of obtained differential metabolites and related metabolic pathways. Finally, we constructed the binding modes of molecular docking of catechin and related proteins to verify their structure–activity relationships.

## 2. Materials and Methods

### 2.1. Materials

Catechin was purchased from Solarbio Science & Technology Co.LTD (Beijing, China). Lipopolysaccharide (LPS) was purchased from Yuanye Bio-Technology Co.LTD (Shanghai, China). Macrophage RAW264.7 cells stored in liquid nitrogen were purchased from Jinan University (Guangzhou, China). Dulbecco Modified Eagle Medium (DMEM) was purchased from Thermo Fisher Scientific Inc (Thermo Fisher, Waltham, MA, USA). Phosphate buffer solution (PBS), penicillin-streptomycin and fetal bovine serum (FBS) were supplied by Gibco Life Technologies (Grand Island, NY, USA). Cell Counting Kit-8 (CCK-8) whose product code was CK04 was purchased from Dongren Chemical Technology Co. LTD (Dongying, China). Nitric oxide (NO) kit whose product code was S0021S was purchased from Beyotime Biotechnology Co. LTD (Shanghai, China). Aspirin was purchased from Solarbio Science & Technology Co.LTD (Beijing, China). Other mentioned chemical materials were all purchased from Guangzhou Chemical Co. LTD (Guangzhou, China). All other reagents used in our research were of analytical grade.

### 2.2. Anti-Inflammatory Effect

#### 2.2.1. Cell Culture

The detailed steps of cell culture have been reported [21]. We offered a humidified atmosphere (5% CO_2_, 37 °C) to culture macrophage RAW264.7 cells which were grown and reproduced in DMEM supplemented with 10% (*v*/*v*) FBS and 1% (*v*/*v*) penicillin-streptomycin. We continued further experiments when the sterile tissue culture flasks were filled with cells.

#### 2.2.2. CCK-8 Assay

The evaluation of the cell viability of macrophage RAW264.7 cells used a CCK-8 assay. The cells were hatched in a humidified atmosphere with 5% CO_2_, 37 °C for 24 h before 100 μL/well of them were adjusted in 96-well plates at a density of 3.0 × 10^4^ cells/mL. Then, 10 μL of catechin was added at the final concentrations of 0, 10, 20, 40 and 80 μg/mL (3 biological replicates per concentration). After another 24 h of incubation, the solution in each well was discarded, and 100 μL of DMEM and 10 μL of CCK-8 solution were added per well. The absorbance of the cultured samples was recorded using a microplate reader at 450 nm after 2 h incubation.

#### 2.2.3. NO Assay

The NO assay of RAW264.7 cells through using commercial Kits could evaluate anti-inflammatory activity. First, 1 mL/well of RAW264.7 cells with a density of 1.0 × 10^4^ cells/mL in 24-well plates were cultured for 24 h at 37 °C. Then, the supernatant was discarded. Meanwhile, 50 μL of catechin at the concentrations of 0, 10, 20 and 40 μg/mL and 50 μL of aspirin (40 μg/mL) were added per well after the addition of 50 μL of LPS (2.5 μg/mL) (3 biological replicates per concentration). Aspirin, one of the anti-inflammatory drugs, is the positive control group [22]. After another 24 h incubation, 50 μL of Griess Reagent I and Griess Reagent II within the NO kit were added per well, respectively. The absorbance of the cultured samples was recorded by using a microplate reader at 540 nm after 2 h incubation.

### 2.3. Network Pharmacology Analysis

Network pharmacology can be applied to preliminarily predict the potential targets, pathways and mechanisms of the anti-inflammatory activity of bioactive components [23]. Detailed analytical methods of network pharmacology have been reported [24]. The potential targets of catechin were obtained by the Traditional Chinese Medicine Systems Pharmacology (TCMSP) database, the Encyclopedia of Traditional Chinese Medicine (ETCM) database [25] and the SuperPred database. Meanwhile, we used the databases of the Online Mendelian Inheritance in Man (OMIM), DrugBank, the Therapeutic Target Database (TTD), the Pharmacogenetics and Pharmacogenomics Knowledge Base (PharmGKB) and GeneCards to select the inflammation-related genes through the keyword of “inflammation”. Then we screened the common targets of catechin against inflammation according to a Venn diagram manufactured by the Venn website. Furthermore, the protein–protein interaction (PPI) network model with species set to “Mus musculus [10090]” was constructed based on the STRING database online platform [26]. Finally, Gene Ontology (GO) enrichment and Kyoto Encyclopedia of Genes and Genomes (KEGG) pathway were analyzed by the Database for Annotation, Visualization and Integrated Discovery (DAVID) [27] and we constructed the visualization of network relationships which was beneficial for further correlation analysis through Cytoscape software (version 3.9.0, Open Source Initiative, Palo Alto, CA, USA).

### 2.4. Metabolomic Analysis

The metabolomic analysis on macrophage RAW264.7 cells was determined via a standard metabolic operating procedure [28]. We set up three groups: model group (BT), control group (BC) and positive control group (BP). Macrophage cells in BT, BC and BP groups were processed for total protein extraction. RAW264.7 macrophage cells were cultured in 40 mm plates at 1 × 10^7^ cell/plate. The positive control group of LPS samples was added at the concentrations of 2.5 μg/mL for 24 h. Compared with the positive control group, the model group of catechin samples was added at the concentrations of 20 μg/mL for 24 h (3 biological replicates per group). Subsequently, we added the mixture (methanol: acetonitrile: water = 2:2:1, *v*/*v*) to the cells, and then using ultrasonication homogenized the samples for 30 min at 4 °C. The samples were centrifuged for 15 min (14,000× *g*, 4 °C) and placed for 1 h at −20 °C to remove the protein. The supernatant was collected and then dried in a vacuum centrifuge. Before the LC−MS analysis, samples were dissolved in acetonitrile/water (1:1, *v*/*v*) solvent. Data acquisition and analysis were performed with XCMS software (version 3.5.1, The Scripps Research Institute, San Diego, CA, USA). In order to normalize the acquired metabolites, pareto-scaled principal component analysis (PCA), partial least-square discriminant analysis (PLS-DA) and orthogonal partial least-square discriminant analysis (OPLS-DA) were performed [29]. The fold change (FC), variable importance projection (VIP) and *p*-value produced by OPLS-DA were applied to discover the contributable variable for classification.

### 2.5. Molecular Docking

The specific methods of molecular docking have been reported [30]. Firstly, we found the 2D structure of catechin on the PubChem website and transferred it to the 3D structure through ChemOffice software (version 2019, Cambridge Soft, Cambridge, MA, USA) with the optimization conditions of minimum free energy. Moreover, through the uniport database, we converted gene names to protein names which could be used to download their 3D structures on the Protein Data Bank (PDB) database [31]. Secondly, we deleted the water molecules and small molecule ligands, added hydrogen, and determined the active pocket in the protein 3D structure through Auto Dock Tools. Finally, we obtained 20 molecular docking models of catechin and proteins via Vina software (version 1.2.3, The Scripps Research Institute, San Diego, CA, USA) and chose the best one with the condition of minimum free energy.

### 2.6. Statistical Analysis

Results were represented as the means ± SD for each experimental group. The data were analyzed by one-way ANOVA followed by a Student’s *t*-test with SPSS 20.0 to compare the control and treatment groups; *p* < 0.05 was considered as statistically significant.

## 3. Results

### 3.1. Anti-Inflammatory Activity of Catechin

**Figure 1:**
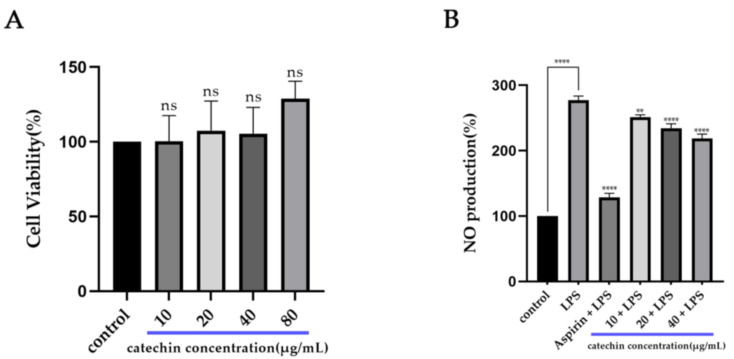
Anti-inflammatory effect of catechin on RAW264.7 cells. (**A**) Cell viability. NS means no significance. (**B**) NO production. **** *p* < 0.0001, other groups vs. control group, aspirin (40 μg/mL) adding group vs. LPS (2.5 μg/mL), and catechin (20 μg/mL and 40 μg/mL) adding groups vs. LPS (2.5 μg/mL); ** *p* < 0.01, catechin (10 μg/mL) adding group vs. LPS (2.5 μg/mL).

In our study, we measured the cytotoxicity of catechin on macrophage RAW264.7 cells by using the CCK-8 kit (Figure 1A). Compared with the control group, the viability of RAW264.7 cells added with catechin presented an increasing trend above 100%. Therefore, we could conclude that catechin had no cytotoxicity within the concentration range (10 μg/mL–80 μg/mL) used and catechin might promote the proliferation of macrophage cells. The production of NO can be used to evaluate the inflammatory state in cells. The result of the NO assay (Figure 1B) showed that the production of NO in the group with the addition of 2.5 μg/mL of LPS had a significant increase (*p* < 0.0001) which was about three times that of the control group. It indicated that cell inflammation was successfully induced. Compared with the LPS group, the production of NO had a significant decrease (*p* < 0.0001) after the addition of catechin. Meanwhile, the production of NO also had a significant decrease (*p* < 0.0001) after the addition of aspirin (40 μg/mL). Thus, we concluded that catechin had an obvious effect on stimulating macrophages to release NO in a dose-dependent manner (10 μg/mL–40 μg/mL) within the using concentration range. These results were also consistent with the existing research [32].

### 3.2. Potential Targets and Pathways of Catechin against Inflammation

**Figure 2:**
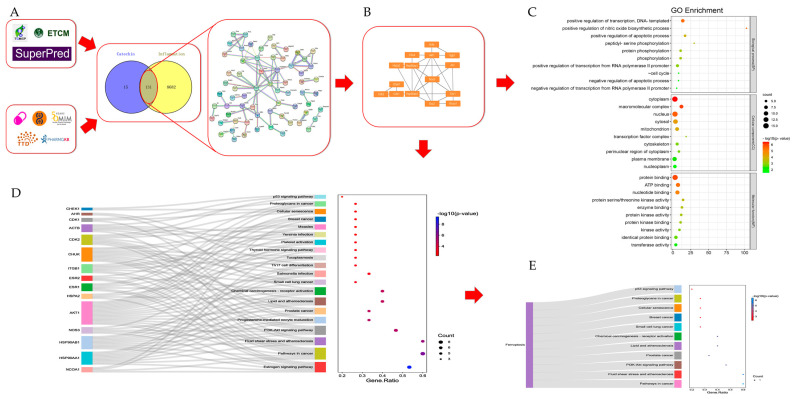
Network pharmacology analysis of catechin on inflammation. (**A**) Potential targets and PPI network. (**B**) 15 core targets. (**C**) Bubble diagram of GO enrichments. (**D**) Sankey diagram of KEGG pathways. (**E**) KEGG pathways related to ferroptosis.

Based on the above databases, we found 146 potential targets of catechin and 8813 genes related to inflammation (  Supplement Figure S1), and their integration was shown in a Venn diagram (Figure 2A). Then, 131 common targets in the Venn diagram were constructed as a PPI network and 15 core targets of them were selected through the Cytoscape software (Figure 2B) (Table 1). Furthermore, we utilized the DAVID online platform to find the GO enrichments including the biological process (BP), the cellular component (CC) and the molecular function (MF). All of their top 10 pathways (count ≥ 4) are shown in the bubble diagram (Figure 2C) which mainly related to inflammatory regulation, cell apoptosis and protein regulation. Meanwhile, the top 20 KEGG pathways with the condition of a *p*-value under 0.006 are shown in the Sankey diagram (Figure 2D). We found that some KEGG pathways were highly associated with ferroptosis, such as pathways in cancer [33], PI3K-Akt signaling pathway [34], lipid and atherosclerosis [35] and p53 signaling pathway [36] (Figure 2E). Thus, we speculated that ferroptosis might be involved in the anti-inflammatory process of catechin. However, all the results of our prediction still need to be further confirmed.

### 3.3. Potential Different Metabolites and Related Metabolic Pathways

**Figure 3:**
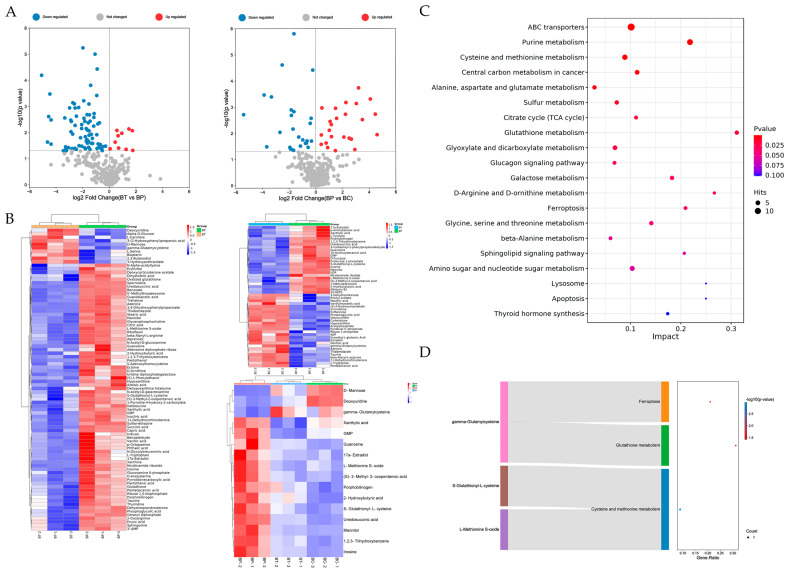
Metabolism analysis of catechin treating on macrophage RAW264.7 cells. (**A**) Volcano plots of differential metabolites in BT vs. BP group and BP vs. BC group. (**B**) Hierarchical cluster figures of differential metabolites. (**C**) Bubble diagram of metabolic pathways. (**D**) Metabolic pathways were related to ferroptosis and the enrichments of differential metabolites.

We ensured that the metabolic results were reliable according to the score plots of PCA, PLS-DA and OPLS-DA in both positive and negative ion modes [37,38] ( Supplement Figure S2). We also sharply noticed the distribution of up-regulation and down-regulation of the differential metabolites in the BT vs. BP group and the BP vs. BC group through the volcano plots (Figure 3A). Then we carried out the hierarchical cluster analysis to further clarify the differential metabolic components (Figure 3B). The results showed that the color contrast between groups was obvious and the color of the same cluster in the group was similar. Thus, the results of the hierarchical cluster analysis were reliable. We also obtained the significantly differential metabolites with the conditions of VIP > 1 and *p*-value < 0.05. We screened 50 significantly differential metabolites; 24 were up-regulated and 26 were down-regulated in the BP vs. BC group ( Supplement Table S1) and we screened 86 significantly different metabolites; 10 were up-regulated and 76 were down-regulated in the BT vs. BP group (Supplement Table S2). Finally, a total of 16 common and opposite differential metabolites were screened (Table 2). Furthermore, we also performed the KEGG metabolic pathway analysis, and found that 16 different metabolites were enriched in 21 KEGG metabolic pathways (Figure 3C). It was worth noting that there were three pathways closely related to ferroptosis, namely cysteine and methionine metabolism [39], glutathione metabolism and ferroptosis. These metabolic pathways included the differential metabolites of l-methionine-s-oxide, s-glutathionyl-l-cysteine and γ-glutamylcysteine (Figure 3D).

These metabolites are also closely related to ferroptosis. L-methionine-s-oxide is an oxide of l-methionine and its reduction is regulated by the l-methionine-s-oxide reductase. This reductase is controlled by the methionine sulfoxide reductase A (MSRA) which belongs to the methionine sulphoxide reductase [40]. L-methionine is an essential amino acid of mammals and it only can be obtained from the outside [41]. Recent studies have shown that the synthesis of GSH has a connection with the sulfur conversion pathway of methionine metabolism and cysteine can be synthesized from methionine [39,42]. Meanwhile, l-methionine is also a sulfur-containing amino acid. Sulfur and ferrous ions can form iron–sulfur clusters which play an important role in reducing iron accumulation [43]. S-glutathionyl-l-cysteine is the product of glutathionylation of cysteine. The glutathionylation of cysteine is considered to be a protective mechanism against irreversible cysteine oxidation [44]. The expression of the glutathione oxidoreductase which belongs to the Coenzyme A disulfide reductase (CDR) can promote the reduction of s-glutathionyl-l-cysteine [45,46]. γ- glutamylcysteine is mainly synthesized from cysteine and glutamic acid through the γ- glutamylcysteine ligase enzyme (GCL) in organisms [47]. It is a precursor of GSH. It can synthesize GSH through glutathione synthase and adjust the GCL to maintain glutathione in a balanced state according to its concentration [48]. At the same time, it can be obtained by the reduction of bis-γ-glutamylcysteine through the regulation of the γ-glutamylcysteine reductase (GSR) in the glutathione metabolic pathway [49]. Thus, based on the results of network pharmacology and metabolism analysis, we concluded that ferroptosis was highly related to the anti-inflammatory effect of catechin treating RAW264.7 cells.

### 3.4. Binding Modes of Catechin with Related Proteins

**Figure 4:**
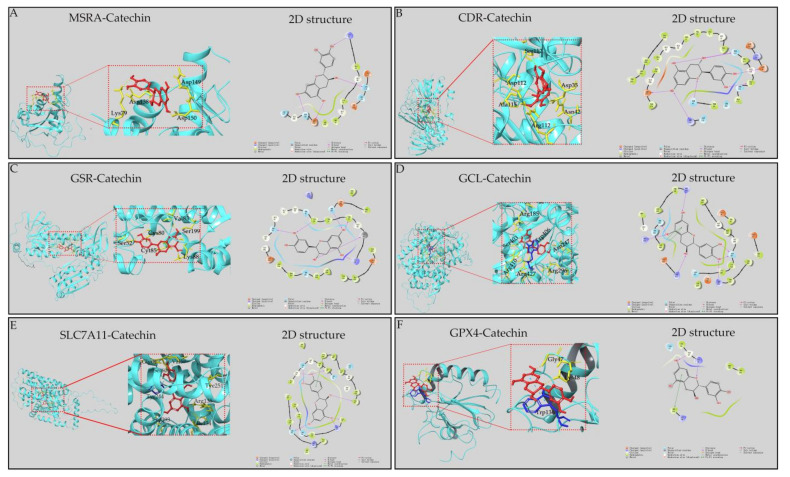
Molecular binding modes of catechin with proteins were presented by 3D and 2D structures. (**A**) Binding modes of MSRA-catechin complex. (**B**) Binding modes of CDR-catechin complex. (**C**) Binding modes of GSR-catechin complex. (**D**) Binding modes of GCL-catechin complex. (**E**) Binding modes of SLC7A11-catechin complex. (**F**) Binding modes of GPX4-catechin complex.

We verified whether catechin interacted with related proteins (MSRA, CDR, GSR and GCL) in three metabolic pathways and ferroptosis-related proteins (SLC7A11 and GPX4) in the anti-inflammatory process by molecular docking. The results showed that all related proteins had a strong affinity with catechin and the combinations were presented by their 3D and 2D structures (Figure 4). Meanwhile, each candidate protein was bound to catechin mainly through hydrogen bonds and Pi–Pi bonds interaction. Furthermore, the low free energy of proteins binding to catechin (binding energy < −30 kcal/mol) demonstrated that the active sites of related proteins were well occupied by catechin and their bindings were highly stable (Table 3). Thus, we could speculate that catechin might interact with the related proteins (MRA, CDR, GSR and GCL) which regulate differential metabolites in three metabolic pathways, and catechin might regulate ferroptosis-related proteins (SLC7A11 and GPX4) in the anti-inflammatory process of catechin.

## 4. Discussion

Based on the above results, we hypothesized the ferroptosis-mediated anti-inflammatory effect of catechin. We further speculated that catechin could activate two metabolic pathways (cysteine and methionine metabolism, and glutathione metabolism) and inhibit the pathway of ferroptosis. This might achieve an anti-inflammatory effect by exerting their synergistic effects in inflammatory diseases to prevent or delay the occurrence of ferroptosis.

As we all know, cells in the body’s innate immune system will produce a large number of ROS such as superoxide and hydrogen peroxide when inflammation occurs. ROS can eliminate pathogens that can cause inflammatory factors in the body [50]. It can also cause oxidative stress such as iron accumulation, the production of various oxides, the increase of lipid peroxidation level, and the differential expression of some genes when it is released in cells for a long time [51]. After l-methionine is ingested from the outside and enters human cells, the sulfur in l-methionine residues will be oxidized to form l-methionine-s-oxide due to the increase of ROS level in cells under the inflammatory environment. This will cause the decrease of l-methionine content. Firstly, the lack of l-methionine will lead to an insufficient supply of sulfur in cells. Sulfur is the key to forming iron–sulfur clusters with ferrous ions in cells. These clusters can reduce iron accumulation to prevent the occurrence of ferroptosis [52]. Secondly, the lack of l-methionine also affects the synthesis of cysteine which is an important precursor for the synthesis of GSH. GSH plays a very important role in antioxidant defense to regulate cell proliferation and apoptosis [53]. It is also an important inducer of GPX4 which is currently considered as an important central inhibitor of ferroptosis [8]. Thus, we speculated that catechin could activate the metabolic pathway of cysteine and methionine to stimulate the activity of MSRA to promote the reduction of l-methionine-s-oxide [54]. It might be one of the effective ways to inhibit ferroptosis to achieve the purpose of anti-inflammation.

S-glutathionyl-l-cysteine, another metabolite in the pathway of cysteine and methionine, may also play an important role in ferroptosis mediating the anti-inflammatory progress of catechin. L-cysteine will be glutathionated to s-glutathionyl-l-cysteine to prevent the occurrence of the irreversible oxidation of l-cysteine under the inflammation environment with a great quantity of ROS. Based on the expression changes of this metabolite in the different groups, we speculated that catechin could activate the activity of CDR to promote the reduction of s-glutathionyl-l-cysteine to l-cysteine.

The differential expression of some enzymes in cells such as SLC7A11 can lead to inflammation. SLC7A11 mainly transports amino acids and is also related to the balance of oxidative stress [55,56]. Some researchers have found that the inhibition of SLC7A11 will result in increased levels of oxidative stress and cell death [57]. Others have also found that the anti-inflammatory effects of resveratrol include up-regulating the expression of SLC7A11 [58]. Meanwhile, the expression of SLC7A11 will be limited when the ferroptosis pathway is activated. The limitation of SLC7A11 can directly affect the transportation and the secretion of cysteine and glutamate, and both of which could synthesize γ-glutamylcysteine according to GCL [47]. The concentration of γ-glutamylcysteine will affect the activity of glutathione synthase which can aggravate ferroptosis and lead to inflammation. Meanwhile, γ-glutamylcysteine can also scavenge free radicals. It can prevent the production of lipid peroxidation and limit ferroptosis [59]. Based on the up-regulation of γ-glutamylcysteine, we speculated that catechin might inhibit the ferroptosis pathway to activate the expression of SLC7A11 to enhance the uptake of cysteine. Moreover, catechin might also activate the activity of the GCL enzyme to accelerate the synthesis of γ-glutamylcysteine to limit ferroptosis and achieve the anti-inflammatory effect.

In addition, the results showed that catechin might also activate the glutathione metabolic pathway. The increasing level of ROS can lead to γ-glutamylcysteine being oxidized to bis-γ-glutamylcysteine under the inflammatory environment. Then, the decrease of γ-glutamylcysteine within the intracellular will cause the influence on the regulation of ferroptosis. Thus, we speculated that catechin could activate the glutathione metabolic pathway to arouse the GSR to promote the bis-γ-glutamylcysteine reduction. It might be an effective way to regulate the ferroptosis in time in the inflammatory environment to avoid the further deterioration of inflammation and achieve the purpose of anti-inflammation.

**Figure 5:**
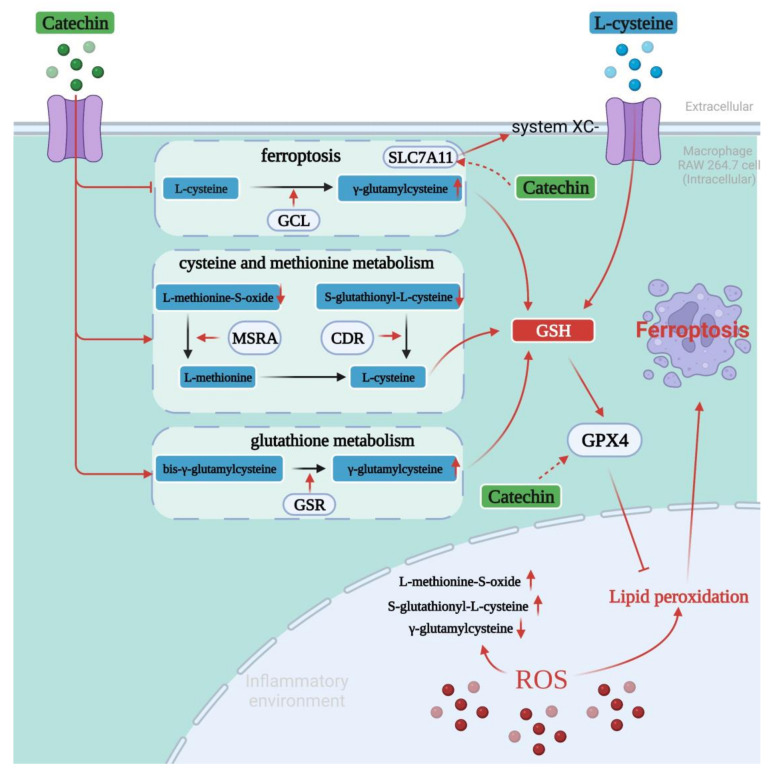
Hypothetical molecular mechanism of ferroptosis mediated anti-inflammatory process of catechin (created with https://app.biorender.com/ (accessed on 18 April 2022)). Lines with red arrowheads represent activation and lines with bars at the end denote inhibition.

Collectively, we mapped the molecular mechanism of the anti-inflammation of catechin mediated ferroptosis on RAW264.7, as shown in Figure 5. We concluded that there was a high probability of ferroptosis in the process of anti-inflammation through the network pharmacological analysis. Further metabolomic analysis showed that catechin could activate two metabolic pathways: cysteine and methionine metabolism, and glutathione metabolism, and inhibit the pathway of ferroptosis. These three pathways were all closely related to ferroptosis. Three key metabolites (l-methionine-s-oxide, s-glutathionyl-l-cysteine and γ-glutamyl cysteine) were regulated in time to prevent or delay the occurrence of ferroptosis which was mainly caused by sulfur deficiency, the limited synthesis of GSH and the increasing levels of lipid peroxidation. This can limit inflammation and complete the anti-inflammatory process. However, it is still not clear whether catechin can affect the activity of reductases involved in the whole anti-inflammatory process and affect factors related to ferroptosis such as GSH and GPX4 directly or not. These still need to be further studied. This study is of great significance in carrying out the activity research of biological components to predict their related targets and pathways with diseases and speculate their molecular mechanism.

## 5. Conclusions

In summary, after the preliminary identification of the anti-inflammatory activity (NO reduction) of catechin on macrophage RAW264.7 cells, we found that ferroptosis might be involved in the anti-inflammatory process of catechin through network pharmacology analysis. Further, three metabolic pathways (cysteine and methionine metabolism, glutathione metabolism and ferroptosis) and three differential metabolites (l-methionine-s-oxide, s-glutathionyl-l-cysteine and γ-glutamylcysteine) closely related to ferroptosis were obtained through metabolomic analysis. Meanwhile, the results of molecular docking also showed that catechin was highly associated with related proteins (MSRA, CDR, GSR and GCL) in three metabolic pathways and ferroptosis-related proteins (GPX4 and SLC7A11). In addition, we also focused on the potential mechanism of catechin mediating ferroptosis to exert an anti-inflammatory effect on RAW264.7 cells. In the future, we need to conduct more experiments such as constructing animal models to further clarify and verify the specific mechanism of anti-inflammatory activity of catechin mediating ferroptosis. This could provide more reliable results for the development of catechin as a new anti-inflammatory drug.

## Figures and Tables

**Table 1:** Information of the core targets.

Gene Name	UniProt ID	Protein Name	Degree Centrality (DC)
Akt1	P31750	RAC-alpha serine/threonine-protein kinase	11
Hsp90aa1	P07901	Heat shock protein HSP 90-alpha	10
Esr1	P19785	Estrogen receptor	10
Hsp90ab1	P11499	Heat shock protein HSP 90-beta	9
Esr2	O08537	Estrogen receptor beta	6
Nos3	P70313	Nitric oxide synthase, endothelial	5
Itgb1	P09055	Integrin beta-1	5
Cdk1	P11440	Cyclin-dependent kinase 1	5
Ncoa1	P70365	Nuclear receptor coactivator 1	5
Ahr	P30561	Aryl hydrocarbon receptor	4
Chuk	Q60680	Inhibitor of nuclear factor kappa-B kinase subunit alpha	4
Cdk2	P97377	Cyclin-dependent kinase 2	4
Chek1	O35280	Serine/threonine-protein kinase Chk1	4
Hspa2	P17156	Heat shock-related 70 kDa protein 2	3
Actb	P60710	Actin, cytoplasmic 1	3

**Table 2:** The information of 16 significantly different metabolites.

Differential Metabolites	VIP (VIP > 1)	*p*-Value (*p* < 0.05)	Fold Change (FC)	BP vs. BC Trend	BT vs. BP Trend
17a-Estradiol	1.23640275	0.02890117	0.63	up ^1^	down ^2^
Xanthylic acid	1.378648746	0.007098714	0.3	up	down
Porphobilinogen	1.228633844	0.041716502	0.66	up	down
1,2,3-Trihydroxybenzene	1.408122971	0.004251857	0.33	up	down
Ureidosuccinic acid	1.479845722	5.71205 × 10^−6^	0.25	up	down
Guanosine	1.381268976	0.041686027	0.29	up	down
GMP	1.387077366	0.002758109	0.1	up	down
**S-Glutathionyl-l-cysteine**	1.350657845	0.011718718	0.39	up	down
Inosine	1.429164585	0.00206919	0.31	up	down
**L-Methionine s-oxide**	1.411921836	0.003672009	0.37	up	down
(S)-3-Methyl-2-oxopentanoic acid	1.226482998	0.033345036	0.24	up	down
2-Hydroxybutyric acid	1.251116465	0.037188342	0.58	up	down
Mannitol	1.449246459	0.001098845	0.2	up	down
Deoxyuridine	1.242857502	0.041777372	1.04	down	up
D-Mannose	1.332811288	0.008474664	3.32	down	up
**γ-Glutamylcysteine**	1.350096846	0.007306674	2.77	down	up

^1^ Up means up-regulation of the differential metabolites. ^2^ Down means down-regulation of the differential metabolites.

**Table 3:** The binding energy for catechin with related proteins.

Compound	Protein	Amino Acid Residues	Docking Score	Binding Energy (kcal/mol)
Catechin	MSRA	Lys79, Asn138, Asp149, Asp150	−5.587	−31.47
Catechin	CDR	Asp35, Asn42, Arg112, Asp112, Ser112, Ala115	−8.428	−55.50
Catechin	GSR	Ser52, Cys80, Val83, Cyt85, Lys88, Ser199	−6.949	−52.12
Catechin	GCL	Arg185, Asn247, Arg296, Ser403, Trp406, Arg410, Arg427	−5.950	−42.67
Catechin	SLC7A11	Val57, Ser60, Gly61, Ile134, Arg135, Tyr244, Tye251, Ser393	−6.229	−44.74
Catechin	GPX4	Gly47, Lys48, Trp136	−3.744	−33.38

## Data Availability

All data are contained in the article and Supplementary Material.

## Supplementary Materials

The following supporting information can be downloaded at: https://www.mdpi.com/article/10.3390/foods11111572/s1, Figure S1: The Venn diagrams of catechin and inflammation; Figure S2: The score plots of the BT vs. BP vs. BC group in positive ion mode (diagram on the left) and negative ion mode (diagram on the right); Table S1: The differential metabolites of BP vs. BC group, log2FC > 0 means up-regulated, log2FC < 0 means down-regulated; Table S2: The differential metabolites of BT vs. BP group, log2FC > 0 means up-regulated, log2FC < 0 means down-regulated.
