# Supplementary material for: Catechin Mediates Ferroptosis to Exert an Anti-Inflammatory Effect on RAW 264.7 Cells
Source: Foods. 2022 May 27;11(11):1572. doi: 10.3390/foods11111572 (PMC9180002; doi:10.3390/foods11111572)
Supplement: Supplementary file 1 [file foods-11-01572-s001.zip › foods-1720092-supplementary.pdf]

Supplementary Data

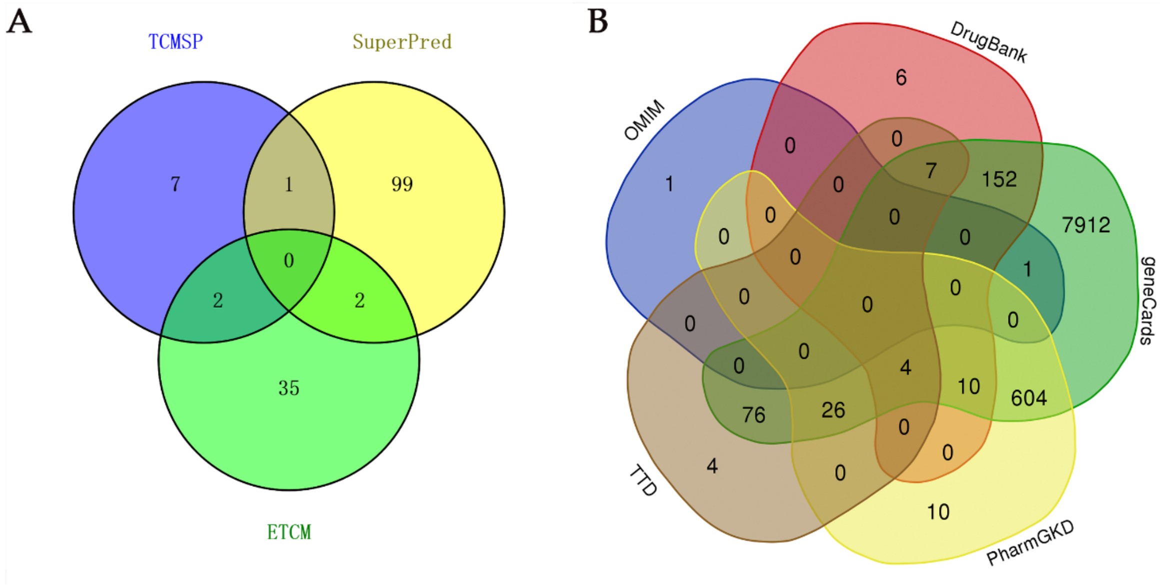

Figure S1. The Venn diagrams of catechin and inflammation. (A) Common targets of catechin from three databases. (B) Common genes of inflammation from five databases.

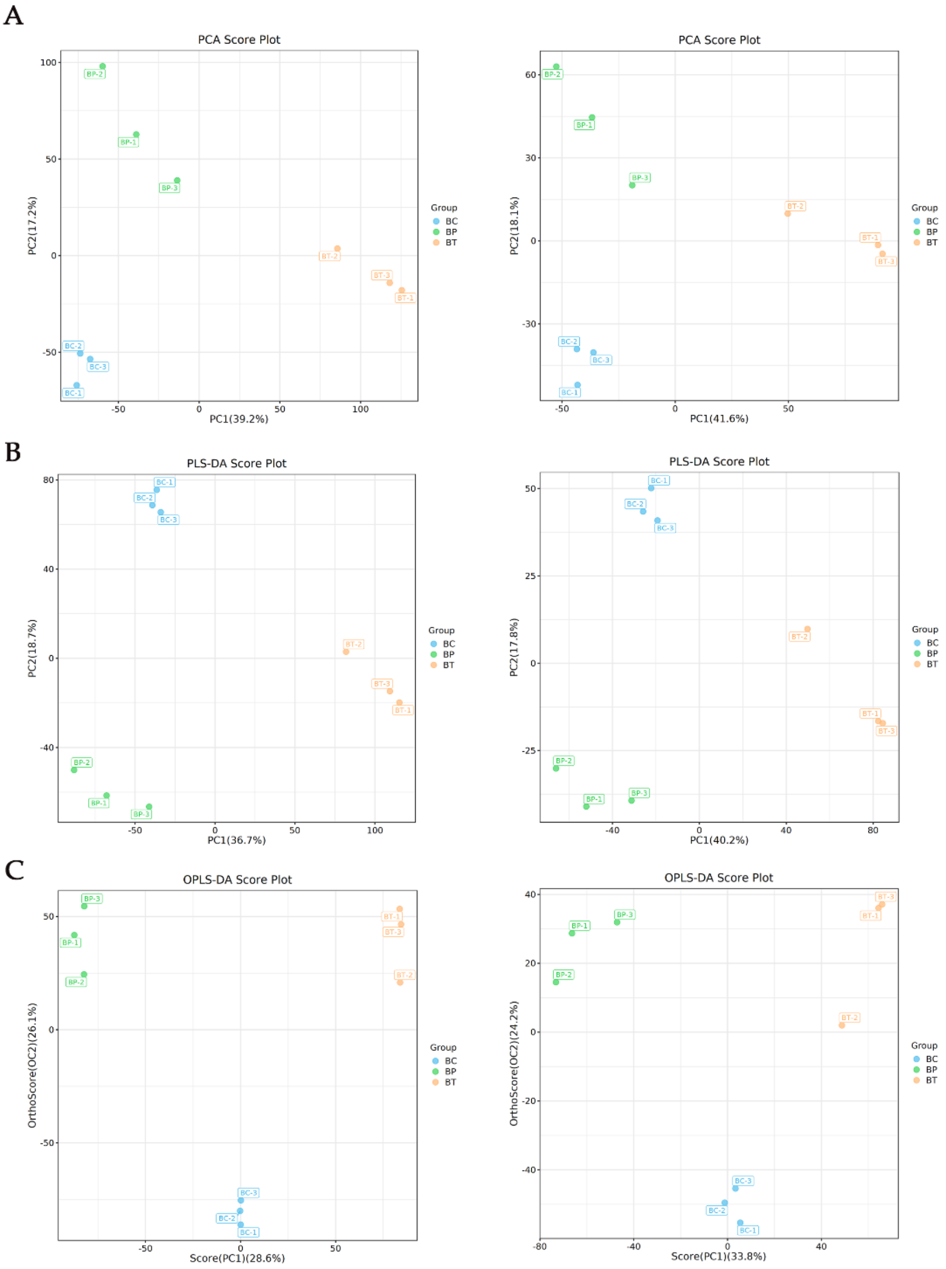

Figure S2. The score plots of BT vs BP vs BC group in positive ion mode (diagram on the left) and negative ion mode (diagram on the right). (A) PCA score plot. (B) PLS-DA score plot. (C) OPLS-DA score plot.

Table S1. The differential metabolites of BP vs BC group. $log_2FC > 0$ means up-regulated, $log_2FC < 0$ means down-regulated.

Differential metabolites	VIP (VIP > 1)	p-value (p < 0.05)	Fold Change (FC)	log_2FC
Ureidosuccinic acid	1.622939952	0.011579856	24.2	4.6
Guanosine	1.604419233	0.001903542	22.09	4.47
UDP	1.632981406	0.000502609	17.14	4.1
1,2,3-Trihydroxybenzene	1.587240304	0.003072613	11.29	3.5
Mannitol	1.644926718	0.000187671	9.34	3.22
Inosine	1.625070515	0.000740986	8.29	3.05
1-Methyladenosine	1.523413438	0.042876353	7.64	2.93
(S)-3-Methyl-2-oxopentanoic acid	1.484410325	0.017104315	6.36	2.67
Nicotinamide ribotide	1.495447078	0.015696048	5.71	2.51
2-Hydroxybutyric acid	1.628813917	0.000677806	4.82	2.27
S-Glutathionyl-L-cysteine	1.601948386	0.001907451	4.73	2.24
3-Carbamoyl-2-phenylpropionaldehyde	1.499814442	0.014239804	4.3	2.11
Aflatoxin B1	1.592934205	0.002730316	2.96	1.57
Porphobilinogen	1.617444166	0.00112163	2.82	1.49
p-Aminobenzoic acid	1.354122971	0.046919326	2.78	1.47
L-Methionine S-oxide	1.502145271	0.013682001	2.26	1.18
20-HETE	1.562477468	0.004712729	2.25	1.17
D-Glucose 1-phosphate	1.568890963	0.003584314	2.12	1.08
GMP	1.53462415	0.007821208	2.03	1.02
D-Fructose	1.540215094	0.03575563	1.86	0.89
17a-Estradiol	1.435137752	0.023987768	1.71	0.78
10-Hydroxydecanoic acid	1.607890252	0.001088382	1.39	0.47
Xanthylic acid	1.510171526	0.011737205	1.34	0.42
L-Tyrosine	1.407529387	0.026862896	1.33	0.41
Deoxyuridine	1.656563031	3.8742E-05	0.86	-0.21
Vanillylmandelic acid	1.467376493	0.020123282	0.8	-0.32
Estradiol	1.591601473	0.002700331	0.79	-0.34
N-methyl-L-glutamic Acid	1.574695238	0.004218076	0.75	-0.41
Maslinic acid	1.401284182	0.034848553	0.67	-0.57
Thiabendazole	1.472241236	0.0198582	0.64	-0.65
ADP	1.448117521	0.024028113	0.61	-0.7
Adenine	1.449073633	0.024433145	0.58	-0.77
Pentadecanoic acid	1.478286822	0.018088158	0.51	-0.97
gamma-Glutamylcysteine	1.42688931	0.031096212	0.45	-1.15
L-Tryptophan	1.50549845	0.014045013	0.42	-1.27
Phosphoglycolic acid	1.662795433	1.57565E-06	0.33	-1.61
Hypoxanthine	1.612515327	0.001504064	0.32	-1.63
Phenyl acetate	1.366488601	0.045028537	0.32	-1.66
Cortexolone	1.54548092	0.006950782	0.31	-1.7

Gentisic acid	1.377678127	0.043383679	0.3	-1.74
beta-Alanyl-L-arginine	1.595043759	0.002083654	0.28	-1.82
11-Dehydrocorticosterone	1.605087412	0.001287282	0.28	-1.83
Ribose 1-phosphate	1.360162206	0.040323585	0.27	-1.89
3-Dehydroshikimate	1.406901012	0.034736894	0.26	-1.94
D-Mannose	1.645585603	2.45446E-05	0.18	-2.5
(S)-4-Hydroxymandelate	1.633549597	0.009201671	0.16	-2.68
D-Ornithine	1.627587318	0.000407769	0.1	-3.31
Pyridoxal 5'-phosphate	1.558356137	0.032960877	0.08	-3.67
Taurine	1.641415239	0.000343953	0.07	-3.86
Acetylphosphate	1.60249694	0.00193708	0.02	-5.4

Table S2. The differential metabolites of BT vs BP group. $log_2FC > 0$ means up-regulated, $log_2FC < 0$ means down-regulated.

Differential metabolites	VIP (VIP > 1)	p-value (p < 0.05)	Fold Change (FC)	log_2FC
L-Serine	1.207780465	0.048357367	3.38	1.76
D-Mannose	1.332811288	0.008474664	3.32	1.73
gamma-Glutamylcysteine	1.350096846	0.007306674	2.77	1.47
Biopterin	1.222408711	0.044009394	2.4	1.26
3-(2-Hydroxyphenyl)propanoic acid	1.331556278	0.010667708	1.92	0.94
L-Carnitine	1.221286198	0.039286437	1.63	0.7
Alpha-D-Glucose	1.318364612	0.013310496	1.58	0.66
3-Hydroxyanthranilate	1.335237432	0.008289068	1.48	0.57
2,3-Butanediol	1.286434531	0.023278651	1.36	0.44
Deoxyuridine	1.242857502	0.041777372	1.04	0.06
Adenosine diphosphate ribose	1.241584086	0.033158743	0.82	-0.28
N-Alpha-acetyllysine	1.25120195	0.034817407	0.72	-0.47
(S)-1-Phenylethanol	1.18987027	0.048959018	0.69	-0.53
Porphobilinogen	1.228633844	0.041716502	0.66	-0.6
17a-Estradiol	1.23640275	0.02890117	0.63	-0.67
Phosphoglycolic acid	1.405589864	0.004238856	0.6	-0.74
2-Hydroxybutyric acid	1.251116465	0.037188342	0.58	-0.78
L-Tryptophan	1.277679598	0.027233062	0.56	-0.84
Pyrrolidonecarboxylic acid	1.395707228	0.004672702	0.55	-0.86
Benzoate	1.475849822	3.69971E-05	0.53	-0.91
N-Acetyl-D-galactosamine	1.234889405	0.040787724	0.53	-0.92
Thiabendazole	1.450088158	0.012117386	0.52	-0.94
Guanidoacetic acid	1.424667447	0.000382199	0.52	-0.94
Spermidine	1.479421002	9.94786E-06	0.47	-1.07
Riboflavin	1.433998294	0.001881779	0.45	-1.14
Stearic acid	1.392371466	0.002637407	0.45	-1.16
Ketoleucine	1.382424651	0.005661913	0.44	-1.17
Erythritol	1.238580504	0.026248187	0.42	-1.25
Adenine	1.438230493	0.016733346	0.41	-1.27
S-Glutathionyl-L-cysteine	1.350657845	0.011718718	0.39	-1.35
Vanillic acid	1.275493472	0.025531366	0.39	-1.36
Succinic acid	1.428031831	0.000156015	0.38	-1.4
L-Methionine S-oxide	1.411921836	0.003672009	0.37	-1.44
Pantothenol	1.359998964	0.043734582	0.37	-1.45
Trehalose	1.432117742	0.002103835	0.36	-1.49
D-Ornithine	1.319424058	0.008639648	0.35	-1.52
Pentadecanoic acid	1.308291198	0.012636853	0.35	-1.52
Hypoxanthine	1.442099769	0.000809902	0.35	-1.53
Thymidine	1.215041766	0.037007677	0.34	-1.55

O-Acetylserine	1.371563109	0.008468973	0.34	-1.56
1,2,3-Trihydroxybenzene	1.408122971	0.004251857	0.33	-1.58
p-Octopamine	1.295007341	0.021203738	0.33	-1.58
Benzaldehyde	1.278786865	0.025487479	0.32	-1.65
beta-Alanyl-L-arginine	1.343111929	0.007928358	0.32	-1.66
Inosine	1.429164585	0.00206919	0.31	-1.67
Oxidized glutathione	1.283605121	0.018860598	0.31	-1.68
Phthalic acid	1.295249967	0.021243012	0.3	-1.73
Xanthylic acid	1.378648746	0.007098714	0.3	-1.73
Guanosine	1.381268976	0.041686027	0.29	-1.79
Glutathione	1.337756731	0.014489726	0.29	-1.8
Ectoine	1.400688248	0.003740785	0.28	-1.84
2-Oxoarginine	1.243893863	0.038422295	0.28	-1.85
11-Dehydrocorticosterone	1.386721503	0.002852159	0.27	-1.9
Glycerophosphocholine	1.46023447	0.000607372	0.26	-1.92
Ureidosuccinic acid	1.479845722	5.71205E-06	0.25	-1.98
1-Pyrroline-4-hydroxy-2-carboxylate	1.395738115	0.004950729	0.24	-2.07
(S)-3-Methyl-2-oxopentanoic acid	1.226482998	0.033345036	0.24	-2.07
3,4-Dihydroxyphenylpropanoate	1.466140827	0.007015916	0.24	-2.08
Dehydroepiandrosterone	1.292416551	0.015907283	0.22	-2.16
Taurine	1.265105248	0.032100424	0.21	-2.27
Mannitol	1.449246459	0.001098845	0.2	-2.3
N-Acetyl-D-glucosamine	1.389311038	0.002359598	0.18	-2.47
3'-AMP	1.19906235	0.043677382	0.18	-2.48
Nicotinamide riboside	1.440733938	0.001151841	0.17	-2.57
Geranyl diphosphate	1.400317948	0.005329931	0.17	-2.57
Sphingosine	1.241339286	0.040317441	0.16	-2.6
Citric acid	1.414209745	0.000799808	0.16	-2.62
Capric acid	1.410583445	0.000725976	0.15	-2.74
Pantothenic acid	1.309566291	0.011453971	0.15	-2.75
Dihydrofolic acid	1.205345815	0.04140462	0.14	-2.8
Azelaic acid	1.368099437	0.00374816	0.14	-2.85
Ribose 1,5-bisphosphate	1.329214813	0.009306016	0.14	-2.88
Uridine diphosphategalactose	1.363741751	0.00335942	0.13	-2.89
Isocitric acid	1.449083607	0.001141105	0.13	-2.98
N-Glycolylneuraminic acid	1.209946927	0.037959763	0.12	-3.06
Erucic acid	1.175141578	0.049837336	0.11	-3.23
Dehypoxanthine futalosine	1.226364242	0.043763118	0.1	-3.26
Xanthine	1.376565592	0.035412126	0.1	-3.28
GMP	1.387077366	0.002758109	0.1	-3.34
Indican	1.188458288	0.049944765	0.09	-3.45
Glucosamine 6-phosphate	1.237910351	0.027760581	0.05	-4.37
Deoxycorticosterone acetate	1.374885226	0.003289652	0.05	-4.37
Sulfamethazine	1.464744836	0.000334869	0.05	-4.46

Alprenolol	1.426019471	0.002444742	0.04	-4.55
S-Adenosylhomocysteine	1.301102605	0.0231619	0.04	-4.64
5'-Methylthioadenosine	1.435792199	6.40747E-05	0.03	-5.08
